# Supplementary material for: Patterns of Intron Gain and Loss in Fungi
Source: PLoS Biol. 2004 Nov 30;2(12):e422. doi: 10.1371/journal.pbio.0020422 (PMC532390; doi:10.1371/journal.pbio.0020422)
Supplement: Table S1 — Also available at http://genes.mit.edu/NielsenEtAl/. (4.3 MB ZIP). [file pbio.0020422.st001.zip › NielsenEtAl/html/75.html]

AN2120.1.NCU05650.1.MG05869.1.FG02233.1


```
 CLUSTAL W (1.82) Multiple Sequence Alignments - Introns Inserted


Sequence 1: NCU05650.1	1094 aa
Sequence 2: MG05869.1	1095 aa
Sequence 3: FG02233.1	1114 aa
Sequence 4: AN2120.1	1093 aa
Alignment Length: 1131 aa
Number Identitical Residues: 277 aa
Alignment Score (without introns) 18974


MG05869.1 	MDRLKLAALLKQCQLP1-------~------DTEQVKSATADLQKNYYPHPQSLLLLLET
NCU05650.1	MDKQTLAALLKESQIP1-------~------DTQRVKAITAELQKNYYVHPECLLLLIEV
FG02233.1 	MDKGKLAQLLEGSQVP1ARIALLS1NLSSPANTEQVKAVTAELQKNFYTKPESLLALVEI
AN2120.1  	MDQQRFLQQLQVVLNP1-------~------TQGNVKEATGILQREYYKHPEALVLLIQV
          	**:  :   *:    *                  .**  *. **:::* :*:.*: *:: 

MG05869.1 	VCVDEDAAIRQLAAVQASRLVNKHWPKVDAGAKGSVREHLVQAVMKEQNAKCRHSESRLI
NCU05650.1	FATHDDLGVRQLAAVQAARLVTKHWGKIPKAQKVQVRQVLLEATLKEQNPKCRHTASRLI
FG02233.1 	SLTHGDAGVRQLASVQALRLIPKFWEKTAQDQRQLARNHLLEGVLKESSAGVRHSLARLI
AN2120.1  	ATGHDDAQLRQLAAVEARSLVGKHWAKVQAGQKPALREQLLRSGVSEANDLVRHSVARVI
          	   . *  :****:*:*  *: *.* *     :   *: *:.. :.* .   **: :*:*

MG05869.1 	ANIASLDFAKGEWKELFDGIFQLSESDNVAQREVGTYLIYSAVESDPRHFNEHLPQLFKT
NCU05650.1	AVIACFDFEEGEWDDLRDIAMNLATNADVNQREVGTYIIFSLIEANPTAF-EDLRKLLEI
FG02233.1 	AGIVSADMENGEGEDFLKHILPLSNNDNVIAREVGSFLLYAILEEDPTHFADNTHDLLKL
AN2120.1  	SAVAKVDLEDGEWADLPNFLMRAADGGNKDERAVSLYILFTILETLGEGFEEKFQDLFTL
          	: :.  *: .**  :: .  :  : . :   * *. ::::: :*     * :.  .*:  

MG05869.1 	LEKCIQDGQSLEVRVNSLMAIGSALMLIDTEDEEDADKVKMVQSLVPAMAGVLRSAVEAG
NCU05650.1	FNHTLRDPQSADVRINTMMSIGACLLMFDPE--EDPQAVNALQTLVPSMVDVLKHVVESG
FG02233.1 	FQSRIEDPQSKEVRINIVRAIGAILMIIEPE--EDEVALKAMQGFVPSLVNILKATVEAE
AN2120.1  	FGKTIRDPESADVRINTLLALSKLAMYLDSA--ENMGPVKAFQDLVPSMVAVLKDAIDQG
          	:   :.* :* :**:* : ::.   : ::.   *:   :: .* :**::. :*: .::  

MG05869.1 	DDEKIKQAFEVLQSFLAYDSSLLGNYLKDLLEFTATIAANKEASEDARTQSLAFLAQAVR
NCU05650.1	DDEKIAQAFDVFQQFLACESSLLGKYLKDLVKLMIDLGGNPQAEDEVRAQALAFLAQTVR
FG02233.1 	DEESYKIVFEVFHSFIAYDSSLLALHLRDLLSFMIELGGNVNAEEDARSQALAFLIQCVR
AN2120.1  	EDDRIMQAFEVFQTLLGFDPALLTVHLKDLVLFMNEIAANTEIDEDTRTQAISFLMQTVQ
          	:::    .*:*:: ::. :.:**  :*:**: :   :..* : .::.*:*:::** * *:

MG05869.1 	YRRMKLMGMGDLVKGLVEQALQILTELDDD--DDDDTTPARLSLTLIAQLSSDLPPRLVM
NCU05650.1	YRRMKIQGMRDVGEELTIKSMVILTEIDDDE-DEDDMSPARSALMLLDQLASDLPPRQVI
FG02233.1 	YRRMKIQGMKDMAAELMVKAMHIVTELDPDD-DEEDLSPARTAISLIDTLSNELPPRQAI
AN2120.1  	YRKLKIQGMR-LGEQLARTAVQIVTELGDAAPNDDDITPARSALGLLDMLAQSLPPSQVV
          	**::*: **  :   *   :: *:**:.   .:::* :*** :: *:  *:..***  .:

MG05869.1 	LPILEQFAKLSSSPDPAQRKAGVIALGISCEGAPDFVNSHFKQILPIVISLLNDSDIEVR
NCU05650.1	VPLLNAFPKFATNSEAGYRKAGILALGTVVEGAPDFIANQIKSIMPHVITLLNDQDVGVR
FG02233.1 	VPLLEQFPHFAGNNDPKYRMASMLALGNAAEGAPDFISTQLQPLLPAIISLLEDADTRVR
AN2120.1  	VPLLNTLGQYFNNANPDYRRAGIMSLGMIVEGAPDFISTQMKEIMPIVLQLLADGELKVR
          	:*:*: : :   . :.  * *.:::**   ******: .::: ::* :: ** * :  **

MG05869.1 	HAALVGLTRLAEEMSEDVAAEHVPLIQALLKNLEAAENSSTDEATKKKNTNIIRSVCGAF
NCU05650.1	HAALVGLSRLADDIASELSPYNEPIMTALVKNLQASMAASNDATQAKKNVEIIRSVCGAL
FG02233.1 	HASLVGLIHLAEEMADEMSSHHEQIISSVLKNLEAASQGPSD----KSNVSIIRCACGAL
AN2120.1  	QATLHAVARLADDLTEDLNRDHEKLMPLLFQNLASAMQESKGE-EEGPTVDIMKAGLSAI
          	:*:* .: :**::::.::   :  ::  :.:** ::   ...      ...*::.  .*:

MG05869.1 	DAMCDGIKPEVMKKFGPELLEPIGKLLSHDDARVKVAAAGAVGAIATAMEQDFVPFFQKT
NCU05650.1	DSMSDGLDAEFMKQYAGELVANIGALIGHEDYKVKVAASGAIGAIAEALGEEFKPYFDNI
FG02233.1 	DTFGDGIDTKIMAQYGPNLIGPMVRLLDHEDFGVKAAAASAIGAIASSMEKSFEPYFKDV
AN2120.1  	DAVVDGLDEKDVAPYQNELVPILHQLFKHPNYRIKGLAAGALGSLASSAGEAFLPFFDDS
          	*:. **:. : :  :  :*:  :  *: * :  :*  *:.*:*::* :  : * *:*.. 

MG05869.1 	MAALGPYMSAKETEEDLTLRGGISDAIGRIAAAVGPEALKPYVQDLMHSTEESLHLDSFE
NCU05650.1	MHALGAYLTIKETEDDLALRSGVCDSVGRIATAVGAQTFQPYVLPLMKSSEEALHLDSTR
FG02233.1 	MTSLGKFVSIKEGEESLDLRSSTCDSLGRIAMAVGSEAFQPYVMDLMTASEEALSLDNPR
AN2120.1  	MHLLQEFATVKDSEEQLDLRASVTDAMGEMSAAAGPERYQAYVEPLMRATEEALHLGHSR
          	*  *  : : *: *:.* **..  *::*.:: *.*.:  :.**  ** ::**:* *.  .

MG05869.1 	LRESTFILWSQLAKVYELDFAPFLDGVFKALLEALELEEEEVTLDLTEEDQAIVGDAKEL
NCU05650.1	LRESSFILWSCLSKVYEKDFSPFLPGVFKGLFHSLELEEEEINLELSEAERAIAGTDEEI
FG02233.1 	LKETSFILWSNLSKVYHEQFDHFLPGVFKGLFSSLELEEEEIEIPGIDASQLGDG---SI
AN2120.1  	LKESTYIFWGAMAKVYGEHFATFLDGAVKGLFNCLEQDDDDLELSLGEAAKDLIG--QEV
          	*:*:::*:*. ::***  .*  ** *..*.*: .** ::::: :   :  :   *  ..:

MG05869.1 	ITAGKRIKIKSPEEDDGLMEDSDDEDLFEDFL----------DSAAAMEKEVAIEVLGDI
NCU05650.1	ITGGKRLKVKANDDED-IMDDEEGED-WEDIG----------ISPEAFEKEVAIEIMGDI
FG02233.1 	VVGGKRVKVKAPENEDDATIANGGDEDWDDIEDMDDLDDLGAVTAVALEQEIALDVLGDV
AN2120.1  	TVAGRKVKVASAEDDDDEPVGEDGEIEDVDLDDEDDWDDITATTPLALEKEIAIEIIGDL
          	 ..*:::*: : :::*.    . .:    *: . .. ..  : :. *:*:*:*::::**:

MG05869.1 	IAHSCGTAEISKHLEKALELIAPLVQHGYEGCRKASVSTLWRAYGRVWQLTEE-ETGVKW
NCU05650.1	ITHSCSGAEIKEYLEKAVEMISPLLEHPYEGCRKAAIATLWRAYARVWQLMEE-ETGSNW
FG02233.1 	ISNSCNSANLETYVEQTIEKVSPFTEHTYEGCRKTAVSTLWRTYARVFQVWEE-GSGVKW
AN2120.1  	VTHTR--SAYLPYFEKTIEMVMPLVEHPYEGVRKATISTMHRSYAMLFTIAEESGQMPKW
          	::::   :    :.*:::* : *: :* *** **::::*: *:*. :: : **:    :*

MG05869.1 	EPGFPPKQTPSVTLQKLGEIVATSTLS-------L2DVVTEINRMVAMILKSCGPAILVG
NCU05650.1	EPGLPLKTQPTVTLVKLGEIVTSATLKVWLEEMDR2AVVTEVNRNVAATLKACGPAILAQ
FG02233.1 	EAGLPAKHTPPASLISMAQALEKATMTIWSDDSER2TVITDINRNIAATLKACGPAVLTC
AN2120.1  	KPGLPLQVEPAKEVKKFGEILMTATIKMWTEEDDR2STVADINRNMAENLRFCGPALIAN
          	:.*:* :  *.  : .:.: : .:*:.   .. .   .::::** :*  *: ****::. 

MG05869.1 	QD-TLSQVITALTSIMTRSHPCQMDLGDEDEDNNVEE-SSEYDWLVIDTALDVVISLALS
NCU05650.1	GD-FMKETISILSTIITRSHPCQQDLGDEDEEQEVEG-SSEYDWLVIDTALDVVIGLAVA
FG02233.1 	KDGFLQELVSVVGLIITRSHPCQQDLGDEEEEPEVDAGSSEYDWLVIDTALDVVVGLAAA
AN2120.1  	ET-TLHNVIQMITDIITKKHPCQLEFAEEDVDAGEET--SEFDWIVVDTALDVVSGMAAA
          	    : : :  :  *:*:.**** ::.:*: :   :   **:**:*:******* .:* :

MG05869.1 	LGPAFQELWKIFEKPIVKFASSNDS-TERSTAVGVIAECIRHMGSTVTPYTERLLKVLVH
NCU05650.1	LGPAFSELWKIFEKPILKFASSEAENLERSTGVGVIAECAANMGATVTPYTGKLMHLLLK
FG02233.1 	LGPSFGELWKIFEKPILRLASSSED-LHRSTAVGTIAEITKYVGEAITPFTESLGQALVR
AN2120.1  	LGESFAELWKVFEKIVLRYAGSTES-IERATAVGVLAECINGMGAASTQFTPAFLKLLVH
          	** :* ****:*** ::: *.*  .  .*:*.**.:**    :* : * :*  : : *::

MG05869.1 	RLSDEDPETKSNAAYGAGQLVLQSNASDKYLPSLQEILSKVEPMLYAQSQENRILDNACG
NCU05650.1	RLSDTDNETKSNAAYATGQLILHSTDSNTYLPQYETILHKLAPMLQIP--EARIKDNASG
FG02233.1 	RLTDPDPLAKSNAAYAIGLVVLNSSDTSKTFPMYPHLWEKLEPLLTVN--EMRMTDNVAG
AN2120.1  	RLSDEDPQTKSNAAYAVGRLIEHSN-SPEVVKEFPTILSRLEQCLHQD--VSRLQDNATG
          	**:* *  :******. * :: :*. :   .     :  ::   *       *: **. *

MG05869.1 	CLSRLMMKHPDRVPIGDFLPNMVSKLPLTDDKDGFEENAPIYECIYKLY1DHSEPTVMSL
NCU05650.1	CLCRMILAHPDRVPLAQVLPALVDLLPLKED---YEENTPVYQCIYKLY~EQGEPTINSL
FG02233.1 	AVSRMMAKNPNNEFVAQALPAVANVLPLQED---YEENAPIFENIYNLY~QHSNPTVEQL
AN2120.1  	CLSRMILKHRESVPIKDVLPVLVNILPLKND---YEENDPLYRMICQMY~KWEDPTIREL
          	.:.*::  : :   : : ** :.. *** :*   :*** *::. * ::* .  :**: .*

MG05869.1 	TPQLIPVFEKVLSPPNEQLTAETRVLVQKIVQQLYQAKPDLFQAHQGVLALAHAG
NCU05650.1	TSKVIPVLESVLSPPEDQLSDETRELVRKLVHQLYSANQALFAANPNALKLAGIV
FG02233.1 	TPQLVGIFEKVLSPPEEQLEPETRQILQQTVQMLYKAKPDLLANNPGLLKLANIQ
AN2120.1  	TPQFLPVFQSVLCGDTDQLEDERRAELIELVKWLNQMQPGAAPWAEQL-------
          	*.:.: :::.**.   :**  * *  : : *: * . :
```
